# Correction: Deep in blue with green chemistry: influence of solvent and chain length on the behaviour of *N*- and *N*,*N*′-alkyl indigo derivatives

**DOI:** 10.1039/d0sc90261f

**Published:** 2020-11-24

**Authors:** Daniela Pinheiro, Marta Pineiro, Adelino M. Galvão, J. Sérgio Seixas de Melo

**Affiliations:** University of Coimbra, CQC, Department of Chemistry Rua Larga 3004-535 Coimbra Portugal sseixas@ci.uc.pt; Centro de Química Estrutural, Departamento de Engenharia Química, Instituto Superior Técnico (IST), Universidade de Lisboa Av. Rovisco Pais 1049-001 Lisboa Portugal

## Abstract

Correction for ‘Deep in blue with green chemistry: influence of solvent and chain length on the behaviour of *N*- and *N*,*N*′-alkyl indigo derivatives’ by Daniela Pinheiro *et al.*, *Chem. Sci.*, 2020, DOI: 10.1039/d0sc04958a.

The authors regret that incorrect compound names and values are reported in Table 5 of the original article. The corrected Table 5 is shown below.

**Table:** Comparison of the fluorescence decay times, *τ*_*i*_, obtained from the ultrafast time-resolved TA studies and from the ps-TCSPC technique for N,N′C_1_Ind and N,N′C_2_Ind in *n*-dodecane and 2MeTHF at *T* = 293 K

Compound	Solvent	fs-TA		TCSPC	
		*τ*_1_ (ps)	*τ*_2_ (ps)	*τ*_1_ (ps)	*τ*_2_ (ps)
N,N′C_1_Ind	*n*-Dodecane		2387		2690
	2MeTHF	1.3	76		76.6
N,N′C_2_Ind	*n*-Dodecane	129.9	1078	309	1064
	2MeTHF		51		62.4

The Royal Society of Chemistry apologises for these errors and any consequent inconvenience to authors and readers.

## Supplementary Material

